# Accurate Prediction of Hydration Sites of Proteins Using Energy Model With Atom Embedding

**DOI:** 10.3389/fmolb.2021.756075

**Published:** 2021-09-20

**Authors:** Pin Huang, Haoming Xing, Xun Zou, Qi Han, Ke Liu, Xiangyan Sun, Junqiu Wu, Jie Fan

**Affiliations:** ^1^ College of Life Sciences, Beijing Normal University, Beijing, China; ^2^ Accutar Biotechnology Inc., Brooklyn, NY, United States

**Keywords:** machine learning, protein, hydration sites, atom embedding, prediction

## Abstract

We propose a method based on neural networks to accurately predict hydration sites in proteins. In our approach, high-quality data of protein structures are used to parametrize our neural network model, which is a differentiable score function that can evaluate an arbitrary position in 3D structures on proteins and predict the nearest water molecule that is not present. The score function is further integrated into our water placement algorithm to generate explicit hydration sites. In experiments on the OppA protein dataset used in previous studies and our selection of protein structures, our method achieves the highest model quality in terms of F1 score, compared to several previous studies.

## 1 Introduction

### 1.1 Protein Hydration Prediction

Solvation of biomolecules is essential for their functionality, and water molecules are crucial in various biochemical processes, such as bridging secondary structures of proteins, acting as proton donor/acceptors in proton wires, and discriminating ligands at binding sites, all of which require knowledge about positions and orientations of explicit water molecules (Bellissent-Funel et al., 2016). Among these functions, water-mediated protein-ligand interactions are of great interest from the computational side. In an analysis of 392 high-resolution protein structures, 76% of the protein-ligand complexes had at least one bridging water molecule at the interface (Lu et al., 2007). Accordingly, many docking programs have been developed to incorporate explicit water molecules in the docking process and yield prediction results, such as WScore (Murphy et al., 2016) from Schrödinger, Rosetta (Lemmon and Meiler, 2013), AutoDock4 (Forli and Olson, 2012). Better understanding and modeling of this interaction is utilized in structure-based drug designs where drug candidates are modified to replace water molecules in the binding pocket, primarily for entropic gains (Bucher et al., 2018).

In the laboratory, water positions in protein structures are mainly obtained by X-ray crystallography, and crystallographic data have shown that protein structures of a 1 Å resolution contain 66*%* more resolved water molecules than a structure of 2 Å resolution(Maurer and Oostenbrink, 2019). Despite this, over 50*%* of deposited structures in the Protein Data Bank (PDB) database have a resolution larger than 2.0 Å (RCSB, 2020), which indicates plenty of crystalline water molecules are not resolved due to the transient dynamic of water molecules and a lack of local information in the density map. Furthermore, protein structures, either obtained by experimental techniques such as nuclear magnetic resonance (NMR) or predicted through computational tools such as AlphaFold (Senior et al., 2020), provide no information about water molecules.

There is an unmet need for a reliable predictive model for protein hydration that can be integrated into and benefit other modeling and experimental systems. However, how the way to implement such a model, via exploiting a limited amount of experimental data, is still being explored.

### 1.2 Related Works

Many *force field* based methods are proposed, given an abundance of simulation programs that already incorporated some established physical models, with built-in approaches for simulations such as Molecular Dynamics (MD) and Monte Carlo (MC) available. For the prediction of explicit hydration sites, an extra step is needed to analyze and cluster the trajectory or histogram of simulations performed on an equilibrated system comprising a protein macromolecule solvated by explicit water molecules. Examples of MD-based methods are WaterMap (Schrodinger, 2020), which is based on the Inhomogeneous Fluid approach to Solvation Thermodynamics (IFST) (Lazaridis, 1998) and WATSite (Hu and Lill, 2014; Yang et al., 2017) which integrates over a probability density function of water molecules to estimate the entropic change. Both of these methods claim an effective consideration of entropic terms, which are believed to contribute substantially to the free energy change in cases like solvation of cavities (Young et al., 2007). The main disadvantage is the time cost, as MD simulations sometimes struggled to escape local minima and failed to sample the state space efficiently. One attempt to circumvent this problem is an MC-based method called JAWS (Just Add Water moleculeS) (Michel et al., 2009) that employs a grid-based Metropolis Sampling of water molecules to directly estimate the free energy. Results from JAWS are satisfactory for isolated cavities, but are not ideal for rather exposed grids due to convergence issues.

The reference interaction site model (RISM) (Beglov and Roux, 1997) with the Kovalenko-Hirata (KH) closure, or the 3D-RISM (Kovalenko and Hirata, 1999), on the other hand, calculate the 3D solvent distribution function directly via the statistical mechanics-based integral equation of liquids, saving simulation time. The distribution function has been used for hydration-site analysis of biomolecules (Yoshidome et al., 2020), and also has been utilized as an intermediate to yield explicit hydration sites by the combining use of water-placement algorithms such as Placevent (Sindhikara et al., 2012), which iteratively finds maximum points of the distribution function for atom insertion, and GAsol (Fusani et al., 2018), a genetic algorithm that decides the occupancy of selected potential hydration sites. The quality of 3D-RISM results depends on the force field parameters used in its calculations, thus it requires careful parameter choices before being put in predictive purposes for specific systems (Roy and Kovalenko, 2021). Masters et al. (2018) studied the combination of WATsite and 3D-RISM with GAsol and claimed a better prediction by the joint model.

Methods that utilize empirical, ad hoc functions for energy estimation of water molecules have been widely adopted for their rapidity. For instance, one of the first attempts to predict hydration sites of protein, GRID (Goodford, 1985), reported over 30 years ago, evaluates the energy of water molecules at certain grid points by a combination of empirical functions (Lennard-Jones, electrostatic and hydrogen bond). Some individual cases were analyzed in this work using contours of energy isosurfaces as a rough depiction of minima of the energy function in space, but no systematic assessment on the predictive power was performed.

The WarPP (WateR Placement Procedure) (Nittinger et al., 2018) method is built on an empirical score of water molecules based on interaction geometries dedicated to hydrogen bond modeling, which is then parametrized manually through large-scale experimental data. The specially chosen score function is continuously differentiable, thus gradient optimizable. Another method called GalaxyWater-wKGB (Heo et al., 2021) used a generalized Born model that also considers hydrogen bond orientation and distance, more importantly, it also includes the solvent accessibility between a protein atom and a water oxygen atom. This method was tested to have a similar recovery rate (it recovers about 80% of crystallographic waters at the cost of producing seven to eight times the number of water molecules) with methods like 3D-RISM while being 180 times faster.

Recently, a method named Hydramap (Li et al., 2020) was proposed to estimate the energy in “statistical potentials”, which quantifies pairwise interactions between water molecules and atoms of protein by counting the occurrence of atoms of certain types near a crystalline water molecule in experimental data. The resulting density map of the statistical potential is then clustered to predict explicit water sites. Although the mean-field strategy significantly reduced the computational cost, this method falls short of the performance of MD-based methods in high-resolution structures, possibly because a coarse grid is used for the placement of water molecules.

Other than using simulation-based methods, docking-based methods like WaterDock (Ross et al., 2012) are developed. WaterDock directly treats water molecules as ligands and uses the ligand-docking program AutoDock Vina Trott and Olson (2010) to predict the docking position of the water molecule. The updated WaterDock2.0 (Sridhar et al., 2017) includes explicit water sites summarized from MD simulations for each functional group, reporting a lower false positive rate. Another related work that builds on WaterDock and Dowser (Morozenko et al., 2014) is the Dowser++ program (Morozenko and Stuchebrukhov, 2016). Dowser++ takes Dowser’s emphasis on the charge-dipole interactions in energy calculation, fixes issues like crashing water sites, and extends the scope of prediction of WaterDock from near the binding pocket to the whole protein. Although Dowser++ outperforms its predecessors, there is a constant underestimation of the number of water molecules, the reasons of which are speculated to be the limited number of predictions allowed in WaterDock and the independent insertion of water molecules with no water-water interaction considered.

Several attempts to introduce neural networks (NN) into this problem have been reported by Ghanbarpour et al. (2020). However, they were unable to produce prediction results for explicit hydration sites. Instead, a modified U-net architecture has been used to feed an input structure into multiple 3D convolutional layers to generate occupancy values at grid points. The U-net is trained using an input data set derived from the aforementioned WATSite Hu and Lill (2014) analysis of thousands of MD simulations, followed by another fully connected layer that predicts thermodynamic properties from the occupancy values.

## 2 Methodology

Inspired by recent efforts (Schütt et al., 2018) in molecular modeling that utilizes NNs as universal approximators to describe physical interactions, our solution to the protein hydration prediction problem is based on explicit NN modeling of the interactions among water molecules and protein atoms, instead of predicting intermediate occupancy values.

Our method comprises two components: scoring and sampling. In the scoring part, we train a neural network-based scoring function *Score*(***p*** ∣ ***prot***) from protein structures in the publicly available protein data bank (Berman et al., 2000). The scoring function evaluates the environment of an arbitrary position ***p*** in a protein ***prot*** and then predicts the shortest distance between ***p*** and a potential water molecule. In the sampling part, we tackle the end-to-end hydration prediction problem. Given a protein structure without water molecules or only partially hydrated, our algorithm utilizes the trained scoring function and successively places missing water molecules into the protein structure.

### 2.1 Learned Scoring Function

As the primary component of our solution, the scoring function (Score(p∣prot)→R, or *the scorer*) probes a given protein structure ***prot*** for potential missing water molecules, by predicting the Euclidean distance from a position ***p*** to the nearest water molecule that is not in the input protein structure ***prot***.

**FIGURE 1:**
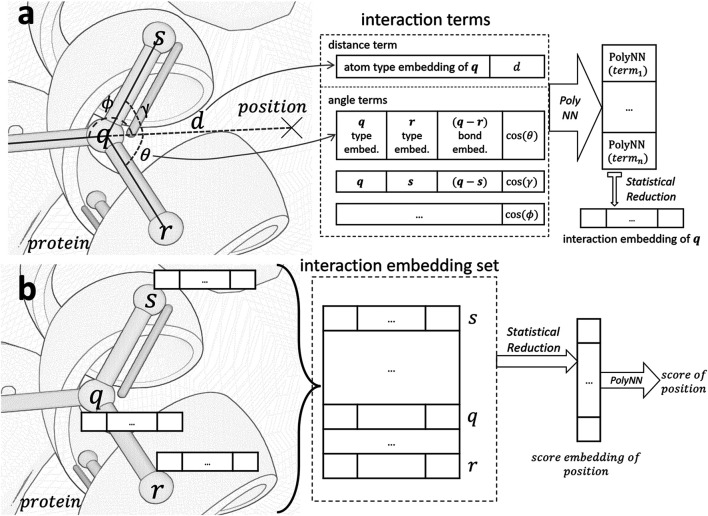
Architecture of the scoring function. **(A)**. Generate the interaction embedding for atom ***q*** in protein from all interaction terms between ***q*** and *position*. **(B)**. Evaluate the score of *position* based on interaction embeddings of all atoms in the receptive field of a 4.0Å radius. PolyNN is our modified version of multilayer perceptron with three fully connected layers.

Figure 1 serves as an illustrative overview of the workflow of our scorer. For a given position ***p***, we calculate interaction embedding for each atom within 4.0Å of ***p***. As shown in Figure 1A, the calculation is based on interaction terms consisting of distance terms and angle terms. These terms are analogues to distance and angle potentials in conventional force fields. We use a statistical reduction method to reduce all embeddings represented by these terms into a single interaction embedding of the atom. After the interaction embedding of each atom is computed, we employ another statistical reduction of these embeddings to obtain the final score, as shown in Figure 1B.

After parametrization of the scorer, our objective is to find positions with scores approaching zero. Apart from modeling atom and bond iterations in the protein structure, we implement a scorer neural network which is continuous and differentiable. This allows us to calculate the derivative of the score over the position and use this as the direction for gradient descent optimization.

#### 2.1.1 Embeddings

**Atom and bond embeddings.** To obtain the embedding for each atom in the input protein, we categorize all atoms that appeared in protein structures into discrete atom types based on their element types, bonded neighbors, and hybridization configurations. An embedding vector is then assigned to each atom type as learnable parameters that will be updated during the training process. Bond type embeddings are similarly categorized, based on the bond types.

**Interaction embedding.** The interaction embedding of an atom ***q*** encapsulates its local information (atom and bond types) and spatial relationship to the position of interest ***p***. Specifically, it is computed over the following interaction terms:

• **Distance term:** Information includes ***q***’s atom type embedding, appended with ***q***’s Euclidean distance to ***p***. Pairwise atom force potentials such as the van der Waals and electrostatic potentials are modeled.
• **Angle term:** Computed for each atom ***r*** bonded to ***q***. We include atom type embeddings of ***q*** and ***r***, concatenated with their bond type embedding, and the angle $cos\left(∠\left(\vec{qp},\vec{qr}\right)\right)$. This term mainly captures the anisotropy of electron distribution, which is critical to the formation of hydrogen bonds.

For each interaction term, the input embeddings and other information are concatenated and fed through a differentiable multi-layer perceptron (section 2.1.2) to obtain an interaction embedding. Interaction embeddings of all atoms in the receptive field are then collected and reduced to a single embedding using the statistical reduction algorithm (section 2.1.3). The reduced embedding is connected to another multi-layer perceptron to compute the final score.

#### 2.1.2 Continuous and Differentiable Multi-Layer Perceptron

The aggregation and reduction function used in previous sections needs to perform vector-to-vector transformations. This is typically implemented using a multi-layer perceptron in neural networks. Since our trained scoring function needs to be used in the subsequent optimization process, it is desirable to be differentiable.

We use a specifically designed layer function for this purpose. This function is called polynomial neural network function (PolyNN), which is a modified version of the multi-layer perceptron. It has three fully connected layers (from input ***x***_**0**_ to output ***x***_**3**_):

$$x_{1}=Swish\left(W_{0}x_{0}+b_{0}\right)$$ (2.1)

$$x_{2}=exp\left(W_{1}\cdot log\left(1+x_{1}\right)\right)-1$$ (2.2)

$$x_{3}=W_{2}\cdot x_{2}$$ (2.3)

Here, ***x***_***1***_ ***,x***_***2***_ are intermediate layers, ***W***_***0***_ ***,W***_***1***_ ***,W***_***2***_ are parameter matrices, and ***b***_**0**_ is a bias vector. The Swish activation function Eq. 2.1 is described and tested by Ramachandran et al. (2017).

Besides being differentiable, the definition allows a continuous modeling of arbitrary algebraic functions rather than conventional multi-layer perceptrons which tends to learn step function-based structures.

#### 2.1.3 Statistical Reduction

The PolyNN network is a one-to-one vector function approximator. Hence for a variable-sized set of vectors such as the interaction embedding set to act as the input of PolyNN, a reduction process is needed. In our work, the statistical reduction is chosen to collect several statistical characteristics as descriptors of the input set, including the summation, average, maximum, and standard deviation values of sets of corresponding components taken from each vector in the input set.

Let the input *n*-dimensional vector set of size *M* be {***x***_1_, ***x***_2_, … , ***x***_*M*_}, and the *j*th element of vector ***x***_*i*_ be ***x***_*ij*_. The statistical reduction layer first calculates the following vectors:

$$sum=\underset{1\leq i\leq M}{\sum }x_{i}$$ (2.4)

$$avg=\frac{sum}{M}$$ (2.5)

$$max=\left\{\underset{1\leq i\leq M}{\max}x_{i1},\underset{1\leq i\leq M}{\max}x_{i2},\ldots ,\underset{1\leq i\leq M}{\max}x_{in}\right\}$$ (2.6)

$$std=\left\{\sqrt{\frac{1}{M}\underset{1\leq i\leq M}{\sum }{\left(x_{i1}-{avg}_{1}\right)}^{2}},\ldots ,\sqrt{\frac{1}{M}\underset{1\leq i\leq M}{\sum }{\left(x_{in}-avg_{n}\right)}^{2}}\right\}$$ (2.7)

The calculated statistical vectors are then concatenated together and a PolyNN layer is then applied to obtain the final output vector of reduction layer ***y***:

y=PolyNN(sum;avg;max;std) (2.8)

### 2.2 Model Training

**FIGURE 2:**
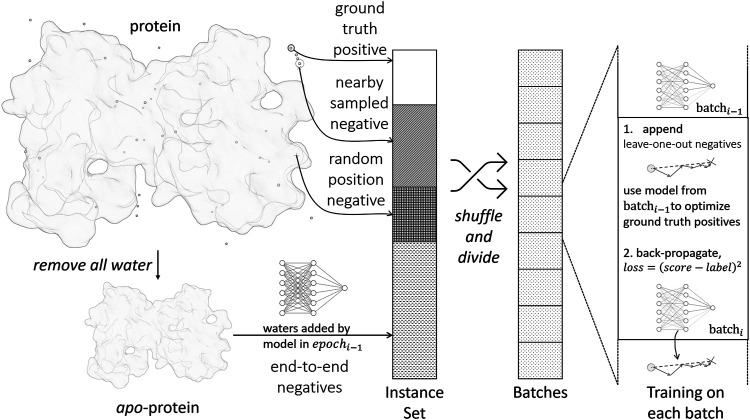
Training process of the scoring function.

The parametrization of the scoring function is accomplished by standard supervised training with the back-propagation algorithm, as illustrated in Figure 2. A training instance consists of a pair {***water***, ***prot***}, where ***water*** is the water position to be predicted, ***prot*** is the environment of the water to be predicted, i.e., protein structure excluding the water. For each training instance, a ***label*** is assigned to denote the distance from the water to its nearest ground truth position. Hence, the training objective is to let *Score*(***water*** ∣ ***prot***) approximate ***label***.

We first extract *static* positive and negative training instances from crystal structures in the following ways:

• Ground truth positives: Positive instances are generated from crystal water positions. The ***label*** is 0 by definition.
• Nearby sampled negatives: For each crystal water, we randomly move its position within 0.8 Å and form a negative instance. The ***label*** is set accordingly.
• Random position negatives: We generate new water molecules and place them randomly in the protein structure. This makes sure the model does not place excess water molecules. We use the full protein ***prot*** in this case without removing any waters from it, and define the ***label*** to be *∞*.

Such simple extracted negative instances are insufficient for training, because most randomly sampled negatives are trivial to identify by the model. To improve the sampling efficiency, we implement *dynamic* negative sampling procedures by generating negative instances on-the-fly during training:

• Leave-one-out negatives: When processing a batch in the training process, we examine each positive instance in the batch. The current model after updating the last batch is used to optimize the position of the ground truth water molecule by gradient descent. The optimized position is appended to the current batch.
• End-to-end negatives: In the final water placement stage, as in section 2.3, we will encounter proteins with partially or wrongly determined environmental waters. To make the model robust in this scenario, we remove all water molecules in a crystal structure and use the water placement algorithm to predict all water positions from scratch. For each predicted water molecule, we find its nearest crystal water position and generate a training instance accordingly.

The loss function for training is defined as:

$$loss=\underset{i}{\sum }weight_{i}\cdot {\left(predict_{i}-Norm\left(label_{i}\right)\right)}^{2}+\lambda |\theta {|}^{2}$$ (2.9)

where the instance index *i* is iterated through the whole minibatch and |*θ*|^2^ represents L2-regularization.

The weights *weight*_*i*_ of the training instances are designed to prioritize training on instances having more interactions with atoms in the protein and less exposure to the bulk solvent, because these water molecules are more likely to be stable and correctly determined by crystallography. The weight is calculated as:

$$weight_{i}=\sqrt{1+1.5\cdot {\text{amino\_count}}_{i}+{\text{water\_count}}_{i}}$$ (2.10)

where amino_count and water_count correspond to the number of amino and water molecules in the instance’s environment, respectively.

**FIGURE 3:**
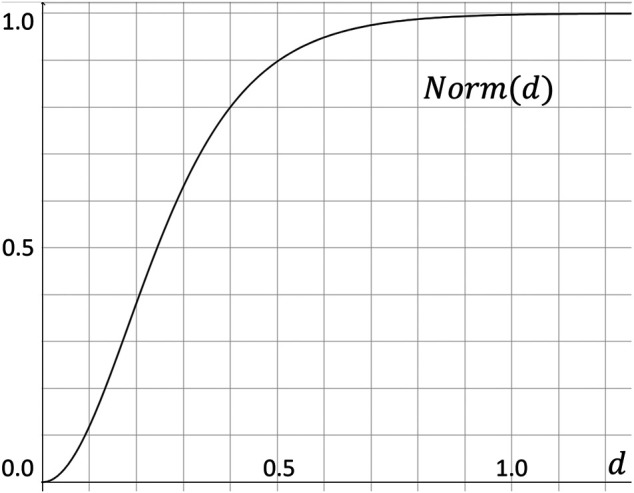
Normalization function *Norm*(*d*), which converts the Euclidean distance *d* to our label value within the [0, 1) range.

From an optimization perspective, the importance of deviations to the model decreases relatively as the absolute distance between the position to be predicted and the ground truth become larger. In order to implement this heuristic, we use a hand tuned normalized function to normalize the labels by a continuous function *Norm*(*d*) that is steep when *d* is relatively small, and become almost constant for *d* ∈ [0.8, + *∞*) (see Figure 3).

$$Norm\left(d\right)={\left(\frac{2}{1+\exp\left(-\frac{5d}{\ln2}\right)}-1\right)}^{2}$$ (2.11)

### 2.3 Water Placement

By design, the property that differs our work from previous studies that handpicked empirical functions or output discrete values like occupancy is the differentiability of our automatically learned function. This differentiability enables subsequent optimization such as gradient descent to be performed. In practice, to attenuate problems such as traps of local minima and suboptimal conformations that arise from the sequential addition of water molecules, algorithms for water placements have been developed as complements to the scorer.

Our water placement algorithm comprises two parts, a placement part and a refinement part. In the placement part, our algorithm probes the current protein structure and finds the location of the potentially missing water molecules; the refinement part combines the water calculated in the previous step with the water molecules already in the protein and optimizes the overall position of all water molecules. Our algorithm runs these two parts alternatively until the placement part cannot add any new water molecule.

#### 2.3.1 Placement

The placement process starts with encompassing the protein with a 3D grid of bounding boxes. The dimension of each bounding box is 0.8 Å, a value that is small enough to ensure the existence of at most one water molecule in each box. After placing the water molecule at the center of each box, gradient descent can be applied to optimize the position.

One can directly calculate scores of these optimized water molecules and keep those with scores better than a predefined threshold. However, there are two major problems in this simple water placement procedure:

1. Because each water is placed and optimized independently, it is possible that the best positions calculated for adjacent grids actually correspond to the same potential water molecule.
2. In crystal structures, there are water molecules that require joint interactions of the protein and other water molecules to stabilize. Such water molecules cannot be probed until all other water molecules that participate in the stabilization are revealed in the input protein structure.

To address these issues, our algorithm uses an iterative placement strategy. In each iteration, we re-optimize water molecules in each box and recalculate their scores, accommodating water molecules added in previous iterations. We then add the water molecule with the best score to the predicted structure. The iteration ends when the best water score is worse than a predefined threshold.

#### 2.3.2 Refinement

The placement step places and optimizes water molecules individually. Therefore it is desirable to optimize all the added water molecules simultaneously. This is easily doable via gradient descent as our scorer is differentiable.

However, solely relying on gradient descent may lead to water molecules trapped in local minima, similar to the behavior seen in force field simulations. To alleviate this problem, we develop a local resampling strategy. Each time a number of adjacent water molecules are selected, and the water molecules in this region are resampled. The resampling procedure first removes water molecules from the prediction results and then tries to add back a subset of these water molecules. The subset with the best score is kept and iteration continues. When the algorithm cannot discover any subset that can be improved, the optimization process ends.

## 3 Experiment Results and Discussions

### 3.1 Evaluation Metric

We evaluate and compare our method with our methods using the using the typical precision and recall metric:

$$\text{precision}=\frac{\text{true positive count}}{\text{number of predicted waters}}$$ (3.1)

$$\text{recall}=\frac{\text{true positive count}}{\text{number of crystal waters}}$$ (3.2)

$$\text{F}1=2\times \frac{\text{precision}*\text{recall}}{\text{precision}+\text{recall}}$$ (3.3)

To count true positives using 3D coordinates of our prediction and crystal water, we set three different cutoffs of the Euclidean distance in our analysis: 0.5 Å, 1.0 Å and 1.5 Å. For each crystal water, at most one predicted water located within the cutoff range is counted as a true positive prediction.

### 3.2 Performance Case Study

In this section, we use the 14 Oligopeptide-binding protein structures (OppA) bound to different KXK tripeptides in the AcquaAlta paper (Rossato et al., 2011) to evaluate the performance of our water placement algorithms. We compare our model with some previous methods: Dowser++, wKGB, HydraMap, GAsol, and WATsite.

We first compare the performances of predicting water positions in ligand binding pockets. In this benchmark, we only consider waters within 4.0Å of both the protein and the binding ligand. The statistical results are shown in Table 1, with the median running times of every method. Our model has large leads on the F1 measure with a moderate running time. It can be seen that other empirical function-based methods, especially wKGB, tend to predict an excessive number of water molecules. Under the 1.5 Å cutoff, wKGB can recall all crystal waters, yet with a much lower precision, compromising the model’s predictive power, which is reflected by its F1 score. This surplus of predicted water molecules suggests the algorithm is oversampling the water molecules and the outputs some clean-up, such as clustering or use of specific water placement algorithms. Among the others, WATsite shows a large lead in terms of performance, which showcases the power of its MD simulation. However, its running time suffers greatly because of the computational heavy MD process. Our neural network model achieves even better performance than WATsite, while maintaining speed comparable to other fast methods.

**TABLE 1:** Results of predicting binding-site waters on the 14-structure OppA dataset (For wKGB, the default output and its output with different score threholds (6,8,10) are all included.

Model	Recall			Precision			F1 score			Median running time(s)
	0.5Å	1.0Å	1.5Å	0.5Å	1.0Å	1.5Å	0.5Å	1.0Å	1.5Å	
Ours	**0.581**	0.847	0.935	**0.386**	**0.654**	0.789	**0.460**	**0.732**	**0.850**	380.3
Dowser++	0.434	0.723	0.854	0.267	0.494	0.633	0.329	0.582	0.720	1625.4
HydraMap	0.179	0.644	0.836	0.069	0.266	0.397	0.099	0.376	0.536	**7.9**
wKGB_all	0.520	**0.905**	**1.000**	0.122	0.228	0.285	0.196	0.364	0.441	265.2
wKGB_6	0.520	**0.905**	**1.000**	0.141	0.268	0.335	0.221	0.412	0.499	265.2
wKGB_8	0.520	**0.905**	0.995	0.151	0.290	0.358	0.233	0.437	0.524	265.2
wKGB_10	0.520	0.899	0.989	0.162	0.308	0.380	0.246	0.457	0.546	265.2
GAsol	0.149	0.465	0.708	0.085	0.307	0.522	0.108	0.367	0.597	1149
WATsite	0.448	0.747	0.843	0.326	0.645	**0.816**	0.375	0.686	0.823	∼ 15000

Best result(s) in each column is(are) in bold font

We also test the harder task of predicting all water molecules within the protein structure. Only the methods capable of predicting non-binding-site waters are compared. The results are shown in Table 2. Again, our method outperforms prior works.

**TABLE 2:** Results of predicting all waters in the 14-structure OppA dataset.

Model	Recall			Precision			F1 score			Median running time(s)
	0.5Å	1.0Å	1.5Å	0.5Å	1.0Å	1.5Å	0.5Å	1.0Å	1.5Å	
Ours	**0.340**	0.550	0.675	**0.218**	0.354	0.437	**0.264**	**0.428**	**0.527**	380.3
Dowser++	0.134	0.261	0.359	0.208	**0.403**	**0.558**	0.162	0.315	0.434	1625.4
wKGB_all	0.278	**0.738**	**0.964**	0.037	0.098	0.134	0.065	0.173	0.235	**265.2**
wKGB_6	0.253	0.638	0.837	0.081	0.205	0.281	0.122	0.309	0.419	**265.2**
wKGB_8	0.227	0.557	0.732	0.109	0.270	0.369	0.147	0.362	0.489	**265.2**
wKGB_10	0.203	0.479	0.622	0.145	0.343	0.461	0.168	0.398	**0.527**	**265.2**

Best result(s) in each column is(are) in bold font

**FIGURE 4:**
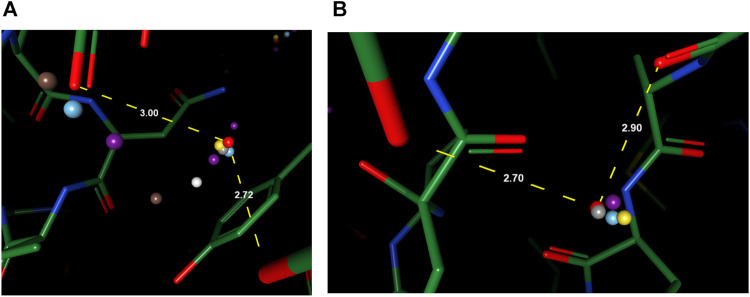
Examples of predicted water molecules of our model in the OppA protein dataset (PDB code: 1B3F), compared to wKGB, Hydramap, and Dowser++ (Red: Ground truth; Yellow: Our prediction; Blue: Dowser++; Purple: wKGB; Brown: Hydramap; Grey: WATsite; White: GAsol.) Prediction results from Hydramap, WATsite, GAsol are binding-site only.

To better understand the prediction results, we analyze several structural scenarios in the OppA protein dataset. Water molecules interacting with several polar atoms in the protein structure are relatively easy for most models, such as water molecules in Figures 4A,B with ideal distances to several polar atoms for the formation of hydrogen bonds. Those easy cases are usually buried, single water molecules in a hydrophilic environment inside the protein, and will be reproduced correctly as long as the model has accurate knowledge of hydrogen bonds such as length and angle distribution.

**FIGURE 5:**
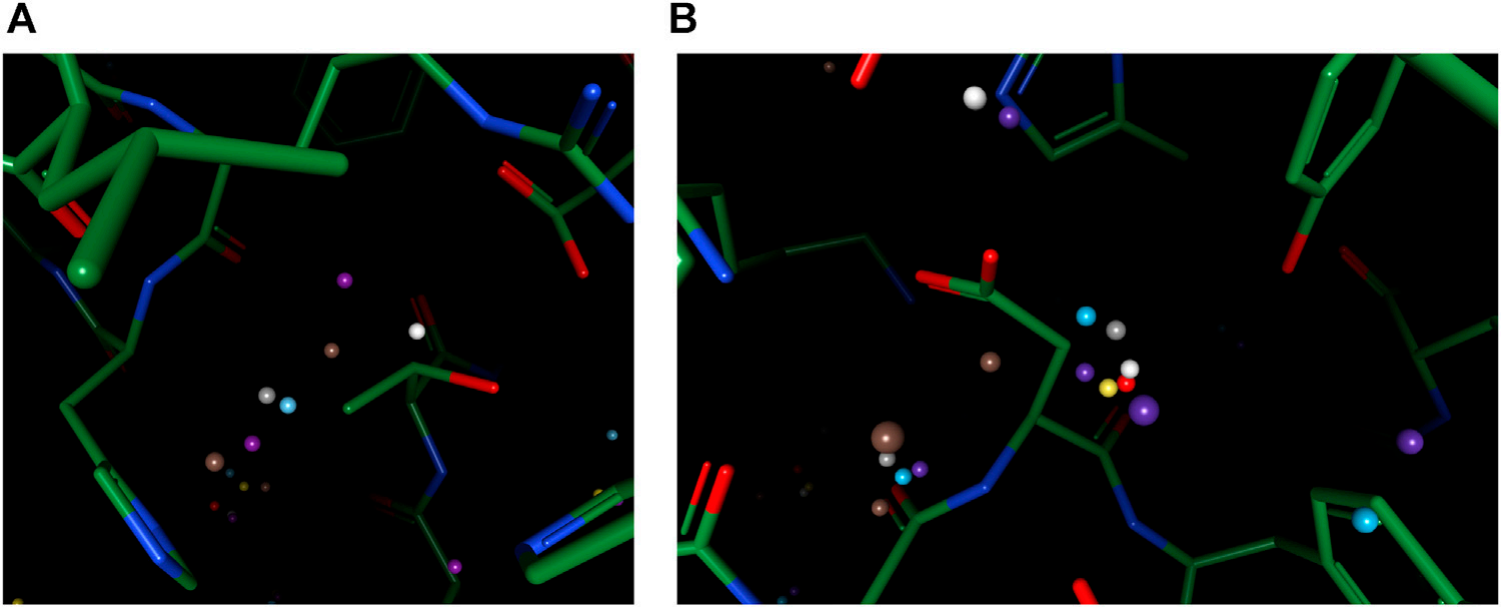
Examples of better prediction made by our model in the OppA protein dataset. PDB ID: **(A)**. 1B3F; **(B)**. 1B5I.

For cases with a mixed environment in terms of hydrophilicity, correct prediction of the mere existence of water molecules can be challenging for many models. For instance, other models predicted the existence of multiple water molecules in Figures 5A,B, while there is zero and one ground truth water within the environment, respectively. Our model, in both cases, outputs the correct number of water molecules, with the position precisely spotted. Water molecules predicted by other models in these two cases seem to be output by the model merely due to their proximity to polar atoms, which suggests the greater difficulty in such environments might arise from the complexity of interactions that necessitate holistic modeling of entropy-enthalpy trade-offs. For example, the addition of a water molecule to a mixed environment may benefit the stability by forming hydrogen bonds with other water molecules or polar atoms yet sacrifice entropic penalties by being too close to hydrophobic moieties.

**FIGURE 6:**
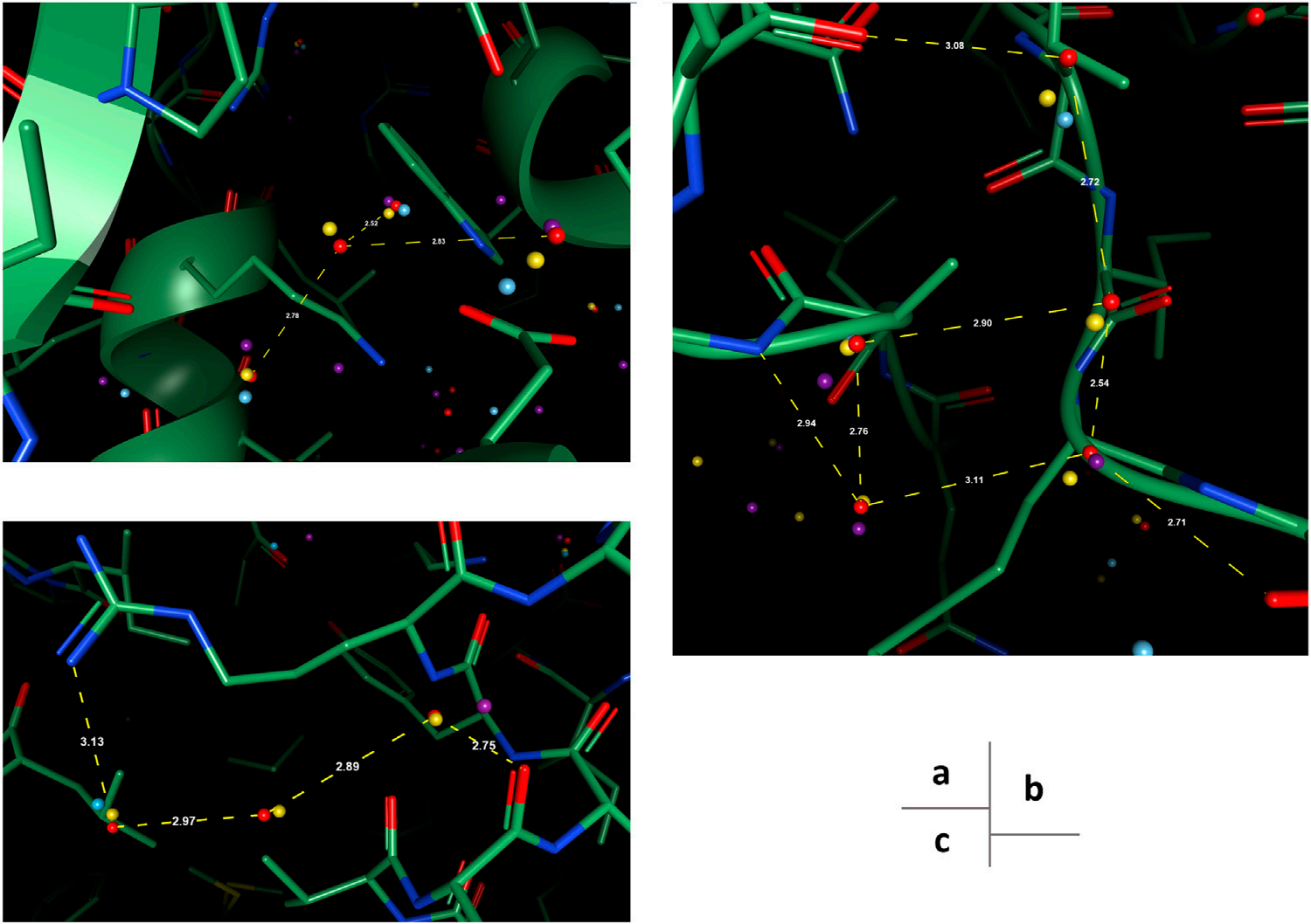
Examples of successfully reproduced water-water interactions in our prediction results, in the OppA protein dataset. PDB ID: **(A)**, **(B)** 1B3F; **(C)** 1B4Z.

To reproduce water-water networks in proteins is more complicated, requiring not only an accurate energy model but also an advanced water placement algorithm. The water molecule reproduced by our model only (at the center of Figure 6A) is an interesting starting example as it bounds to three other “trivial” water molecules that are predicted by all three models, with a rather safe distance with hydrophobic atoms insight. This water molecule, being mostly stabilized by water-water interactions, may be hard to predict if the algorithm cannot iteratively update the environment and uses only the input protein structure for predictions. Two more successful predictions of water networks are given in Figures 6B,C. In both cases, while other models can predict part of the network, which are mainly ones that directly interact with the protein, our model bridges the gap and reproduces the water network precisely.

### 3.3 Large Dataset Benchmark

In this section, we test the algorithms on a large structure dataset comprising 413 high resolutions X-ray structures randomly selected from the RCSB PDB database. Due to time and usage constraints, we only compare our method with Dowser++ and HydraMap. The results are shown in Table 3. Our method shows similar performance as it did in the small dataset, while Dowser++ suffers greatly. This is possibly due to the manually parameterized docking algorithm it is based on, while our neural network-based method is better generalized on a large variety of structures.

**TABLE 3:** Results of predicting all waters by our model and Dowser++, on 413 selected protein structures (The results of Dowser++ are averaged over 380 successfully processed structures).

Model	Recall			Precision			F1 score		
	0.5Å	1.0Å	1.5Å	0.5Å	1.0Å	1.5Å	0.5Å	1.0Å	1.5Å
Our_full	**0.307**	**0.512**	**0.640**	**0.229**	0.384	0.486	**0.256**	**0.427**	**0.537**
Dowser++	0.076	0.155	0.215	0.188	**0.390**	**0.544**	0.104	0.214	0.297

Best result(s) in each column is(are) in bold font

To test the performance on binding-site waters, we remove protein structures without a proper binding ligand from the previous dataset and obtain 100 protein structures. The results are shown in Table 4. In this scenario, Dowser++ performed better compared to the previous task, but our method still holds a clear edge.

**TABLE 4:** Results of predicting binding-site waters by our model, Dowser++ and HydraMap, on 100 selected protein structures (The results of Dowser++ are averaged over 91 successfully processed structures).

Model	Recall			Precision			F1 score		
	0.5Å	1.0Å	1.5Å	0.5Å	1.0Å	1.5Å	0.5Å	1.0Å	1.5Å
Our	**0.389**	**0.621**	**0.755**	**0.240**	**0.390**	**0.490**	**0.283**	**0.455**	**0.559**
Dowser++	0.151	0.294	0.369	0.144	0.313	0.457	0.133	0.275	0.368
HydraMap	0.043	0.314	0.753	0.014	0.083	0.185	0.018	0.122	0.277

Best result(s) in each column is(are) in bold font

**FIGURE 7:**
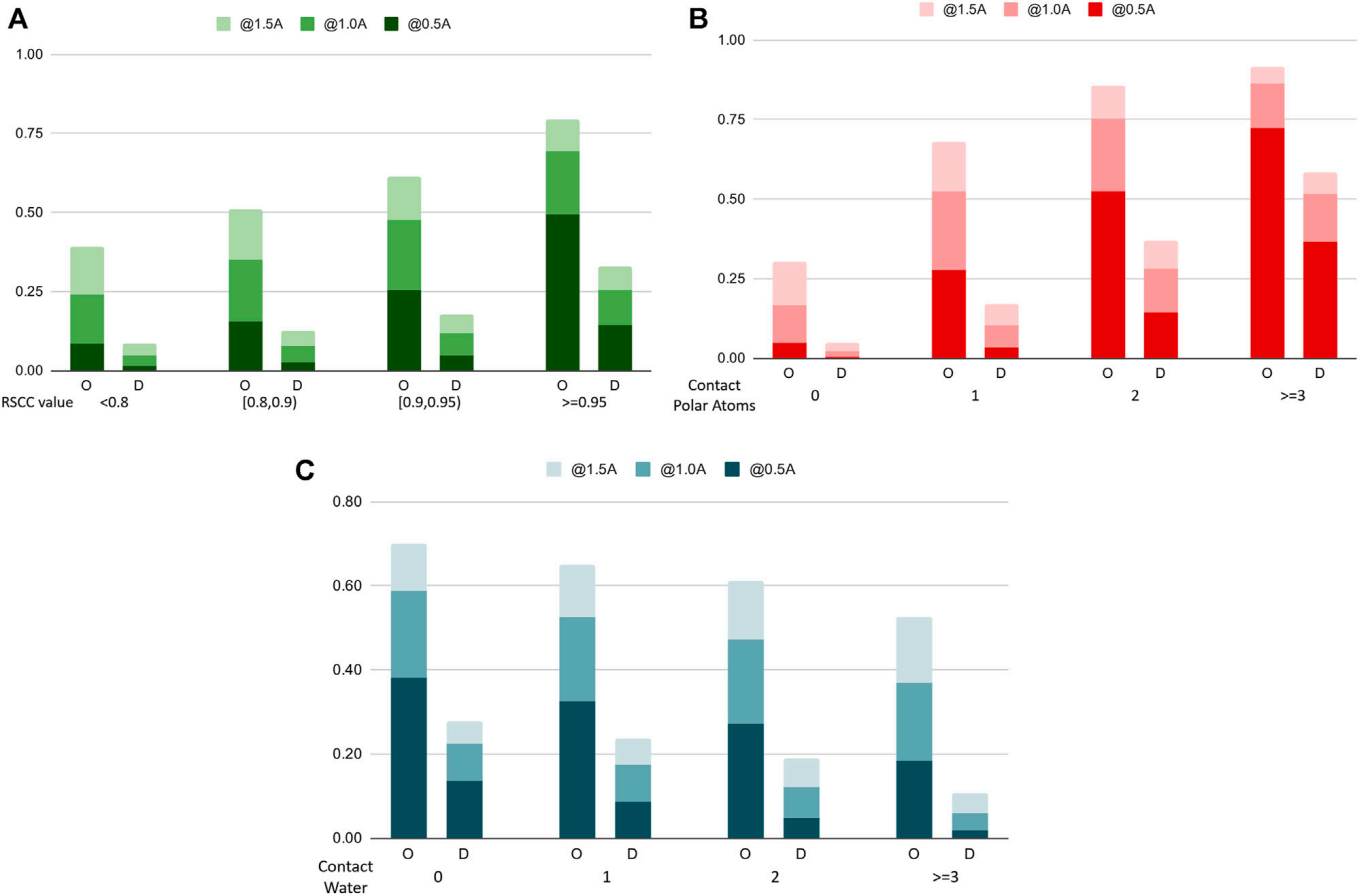
Recall rates of water molecules, categorized by different categorizations of the water molecules **(A)**. The Real Space Correlation Coefficient. **(B)**. Number of polar atoms of the protein nearby. **(C)**. Number of water molecules nearby. O: our model, D: Dowser++.

To further analyze the performance characteristics of the algorithms in terms of different types of water molecules, we categorize water molecules in the benchmark dataset into subsets and compare the recall rate on these sets. Figure 7 shows the comparison chart. In Figure 7A, we categorize the water molecules by their real-space correlation coefficient (RSCC, a common measure used in crystallography to measure the similarity between the model and the experimental density map) of the oxygen atom, and test the recall rate on different RSCC value ranges. The figure shows that water molecules with lower RSCC tend to be harder to predict, which agrees with the fact that RSCC can measure the certainty of the existence of an atom at its location in the model. Lower RSCC corresponds to higher uncertainty of the atom’s position, and may even indicate an incorrectly resolved water molecule at this position, which should not be predicted by a reliable model. In Figure 7B, we find the number of nearby polar atoms of each water molecule and calculated the recall rate for water molecules grouped by the number of polar atom neighbors. The results indicate that without explicit prior domain knowledge, the model successfully learns that polar atoms are highly related to the distribution of water molecules, hence having a very high success rate when the number of polar atoms surrounding a water molecule is high. Figure 7C shows the differences in performance when water molecules are categorized by the number of contacting waters, which can be seen as a measure of the solvent exposure ratio of a certain location.

## 4 Conclusion

Due to the importance of water molecules in protein modeling, many methods for predicting water molecule positions are developed over the years. One major drawback of previous works is the reliance on domain knowledge and explicit parameterization. In this paper, we discuss a novel water placement algorithm using deep learning. We show that without any manual parameterization, the performance of our model surpassed peers by a large margin. Such progress of hydration site prediction is expected to serve other applications as well, such as ligand docking and protein crystal structure refinement.

## Data Availability

Publicly available datasets were analyzed in this study. This data can be found here: https://www.rcsb.org/. Free assessment of our model is available on our Accutar Open Access platform (https://oa.accutarbio.com/).
