# Supplementary material for: Accurate Prediction of Hydration Sites of Proteins Using Energy Model With Atom Embedding
Source: Front Mol Biosci. 2021 Sep 20;8:756075. doi: 10.3389/fmolb.2021.756075 (PMC8488165; doi:10.3389/fmolb.2021.756075)
Supplement: Supplementary file 2 [file DataSheet3.PDF]

frontiers

Supplementary Material

1 PDB LISTS

PDB ID of protein structures used in this study are provided in the supplementary xlsx file.

PDB files with our predicted water positions are also included as separate ZIP files.

2 BINDING SITE FILTERING METHOD

For the 413 protein structures in our test set, a proper ligand is defined to be a non-solvent molecule with its molecular weight within the range of [200,800] Daltons. Also, to avoid ambiguity, there should not be other ligand molecules within 4.0Å of this ligand. Only the ligand with the most heavy atoms is chosen in proteins with multiple ligands, and in cases of a tie, the one with more crystallographic water molecules is chosen.

3 WATER PLACEMENT ALGORITHM

The pseudocode of two critical algorithmic part invoked in our algorithm is described in algorithm 3.1 and algorithm 3.2 respectively, our algorithm will iteratively call AddNewWater() and GlobalAdjust() until the AddNewWater() procedure can not discover any new water molecules.

Algorithm 3.1: Adding new waters

Input: Given protein $\boldsymbol{prot}$
Learned Scoring Function $Score(\boldsymbol{p} \mid \boldsymbol{prot})$
score threshold $\boldsymbol{cutoff}$, a constant value choosed using validation dataset.
Output: Protein $\boldsymbol{prot}$ with additional water molecules predicted by algorithm.

Function `AddNewWater` (*protein* $\boldsymbol{prot}$, *threshold* $\boldsymbol{cutoff}$)
  $\boldsymbol{GP} \leftarrow$ `GenerateGridPointSet` ($\boldsymbol{prot}$)
  while *True* **do**
    $\boldsymbol{candidates} \leftarrow \{\}$
    foreach *candidate point* $\boldsymbol{c} \in \boldsymbol{GP}$ **do**
      $\boldsymbol{c_{opt}} \leftarrow \operatorname{argmin}_{p \in N(c, 0.8\text{Å})} Score(\boldsymbol{p} \mid \boldsymbol{prot})$
      `//` $N(\boldsymbol{c}, \boldsymbol{d})$ `means all points within distance` $\boldsymbol{d}$ `of` $\boldsymbol{c}$
      $\boldsymbol{candidates} \leftarrow \boldsymbol{candidates} \cup \{\boldsymbol{c_{opt}}\}$
    end
    $\boldsymbol{c_{best}} \leftarrow \operatorname{argmin}_{p \in \boldsymbol{candidates}} Score(\boldsymbol{p} \mid \boldsymbol{prot})$
    if $Score(\boldsymbol{c_{best}} \mid \boldsymbol{prot}) < \boldsymbol{cutoff}$ **then**
      $\boldsymbol{prot} \leftarrow \boldsymbol{prot} \cup \{\boldsymbol{c_{best}}\}$
    else
      break
    end
  end
  return $\boldsymbol{prot}$

Algorithm 3.2: Water adjusting algorithm based on iterative local adjustments

Input: protein $\boldsymbol{prot}$, with some already added water molecules.
Learned Scoring Function $Score(\boldsymbol{p} \mid \boldsymbol{prot})$.
Score Cap $\boldsymbol{cap}$.
// $\boldsymbol{cap}$ is a constant obtained from validation set for the total score of waters.
Output: Protein $\boldsymbol{prot}$ with water molecules adjusted to optimum positions.

Function `GetTotalScore` (*protein* $\boldsymbol{prot}$)
  $tot\_score \leftarrow \Sigma_{water w \in p} \boldsymbol{cap} - Score(\text{position of } \boldsymbol{w} \mid \boldsymbol{prot} \setminus \boldsymbol{w})$
  return *tot_score*

Function `LocalAdjust` (*protein* $\boldsymbol{prot}$, *local water set* $\boldsymbol{W}$)
  $\boldsymbol{candidates} \leftarrow \boldsymbol{W}$
  foreach *pair of points* $(\boldsymbol{a}, \boldsymbol{b}) \in \boldsymbol{W}$ **do**
    $\boldsymbol{candidates} \leftarrow \boldsymbol{candidates} \cup \frac{(\boldsymbol{a},\boldsymbol{b})}{2}$ // added midpoint
  end
  $\boldsymbol{candidates} \leftarrow \boldsymbol{candidates} \cup \frac{\sum \boldsymbol{W}}{|\boldsymbol{W}|}$ // added gravity center
  $\boldsymbol{init} \leftarrow \boldsymbol{prot} \setminus \boldsymbol{W}$
  $\boldsymbol{W}_{opt} \leftarrow \boldsymbol{W}$
  $\boldsymbol{score} \leftarrow$ `GetTotalScore` ($\boldsymbol{prot}$)
  foreach *subset* $\boldsymbol{W}_{cand} \subseteq \boldsymbol{candidates}$ **do**
    $\boldsymbol{prot}_{cand} \leftarrow \boldsymbol{init} \cup \boldsymbol{W}_{cand}$
    apply gradient descent optimization for $\boldsymbol{W}_{cand}$ in $\boldsymbol{prot}_{cand}$
    if `GetTotalScore` ($\boldsymbol{prot}_{cand}$) $> \boldsymbol{score}$ **then**
      $\boldsymbol{score} \leftarrow$ `GetTotalScore` ($\boldsymbol{prot}_{cand}$)
      $\boldsymbol{W}_{opt} \leftarrow \boldsymbol{W}_{cand}$
    end
  end
  return $\boldsymbol{W}_{opt}$

Function `GlobalAdjust` (*protein* $\boldsymbol{prot}$)
  // global adjustment of all waters in prot
  $\mathcal{S} \leftarrow$ All 2 or 3 water subset with avg pairwise dist $\leq$ 4Å in $\boldsymbol{prot}$
  while $\mathcal{S}$ *is not empty* **do**
    $\boldsymbol{W}_{old} \leftarrow \text{argmin}_{\boldsymbol{W} \in \mathcal{S}}\, avg\_min\_pairwise\_dist(\boldsymbol{W})$
    $\boldsymbol{W}_{new} \leftarrow$ `LocalAdjust` ($\boldsymbol{prot}$, $\boldsymbol{W}_{old}$)
    if $\boldsymbol{W}_{old} = \boldsymbol{W}_{new}$ **then**
      $\mathcal{S} \leftarrow \mathcal{S} \setminus \{\boldsymbol{W}_{old}\}$
    else
      $\mathcal{P} \leftarrow$ All water subset in $\mathcal{S}$ which contains any water of $\boldsymbol{W}_{old}$
      $\boldsymbol{prot} \leftarrow \boldsymbol{prot} \setminus \boldsymbol{W}_{old} \cup \boldsymbol{W}_{new}$
      $\mathcal{Q} \leftarrow$ All 2 or 3 water subset with avg pairwise dist $\leq$ 4Å in $\boldsymbol{prot}$ which contains any water of $\boldsymbol{W}_{new}$
      $\mathcal{S} \leftarrow \mathcal{S} \setminus \mathcal{P} \cup \mathcal{Q}$
    end
  end
  return $\boldsymbol{prot}$

4 IMPACT OF WATER PLACEMENT ORDER

During water placement, we use an order dependent algorithm to simultaneously solve the collision of water molecules and model the complex water-water interactions. One natural question is how the placement order affects the final performance. We created an alternative algorithm with no score updating

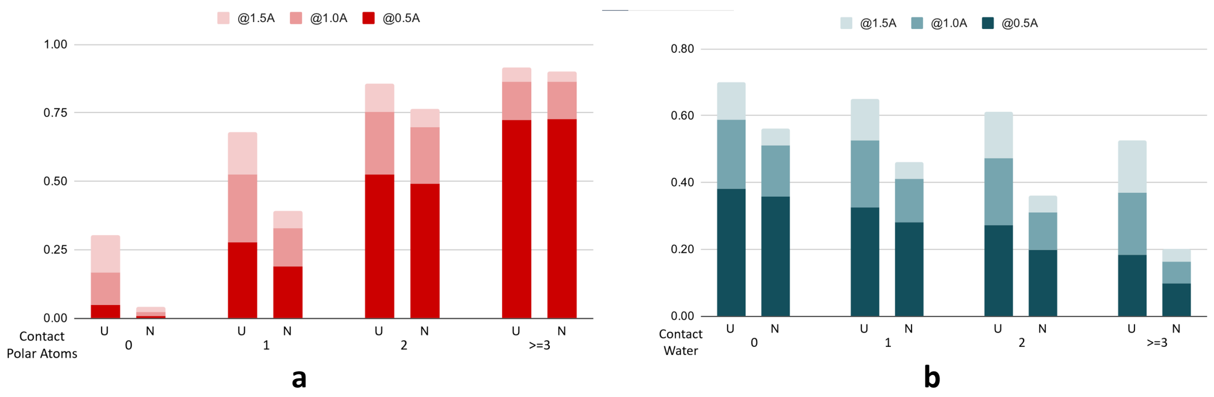

Figure 4.1. The influence of the score update during addition. Recall rates of water molecules categorized by: **a.** Number of polar atoms of the protein nearby. **b.** Number of water molecules nearby. U:Update score during addition; N:No score updates in the addition process.

after every placed water molecule. To avoid duplicated water molecules, we discarded boxes that are within 2Å of already placed water molecules. Experiments with no grid score updates are carried out to have a performance comparison with our final solution (Figure 4.1).

It is illustrated that for water molecules having strong interactions with protein(e.g. having ≥ 3 polar atoms within 3.2Å), the recall rates obtained by the no-grid-score-update approach are almost the same as ones in our final solution. For those with fewer polar atom interactions, the recall rate decreases severely. We also studied the influence of contact waters count, which may reflect the solvent exposure ratio of a certain location. Experimental results showed that water molecules with fewer contact waters were also much easier for our no-update solution to discover. Our algorithm is thus proved to be almost order-invariant for those waters with enough evidence, and the clear deterioration of our no-update approach on waters with fewer interactions also proved the necessity of our iterative updating strategy.

5 PARAMETRIZATION OF THE MODEL

Our scorer model was trained with an Adam optimizer with $\beta_1 = 0.9$, $\beta_2 = 0.999$, $\epsilon = 10^{-8}$ and initial learning rate equal to 0.0001 for 30 epochs, the mini-batch size was equal to 20. There is an automatic learning rate decay that reduces the learning rate linearly after each epoch and eventually to $0.00001(10\%$ of initial learning rate).

The training losses of each epoch is in Figure 5.1.

After inspecting the training curve, we concluded that there is no clear indication of overfitting on both validation loss and average leave-one-out (LOO) optimization distances. The model from epoch 26 was chosen.

There is another important hyperparameter for our water addition algorithm, the threshold of score in water addition. The threshold is set as 0.55 after scrutinizing prediction results of protein structures from the training set and validation set. A larger threshold will slow down the algorithm due to the consequent increase of water molecules that will raise the computational complexity of the adjusting procedure.

Water molecules in the data bank are resolved and determined by various softwares and labs. For the uniformity and quality of instances used in our training process, we redid the ground truth water molecules from density maps. Water molecules are added by iteratively finding optimal density map locations for the addition and update the map to include the newly added water molecules.

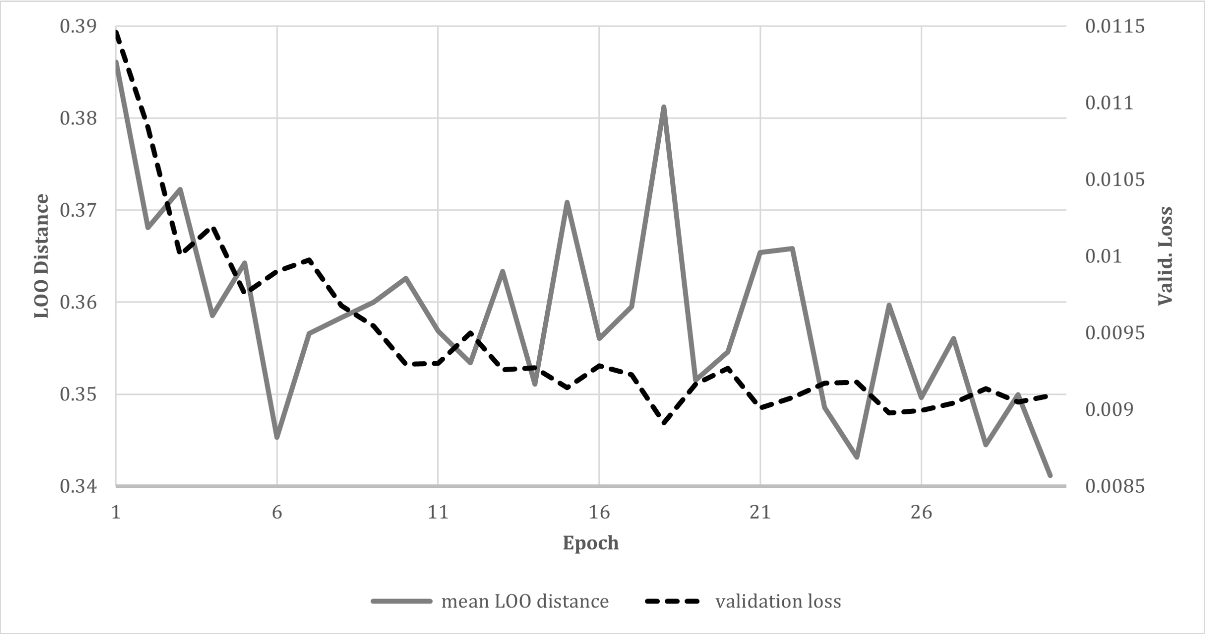

Figure 5.1. Training curve, measured by validation loss and mean leave-one-out (LOO) distance

6 EXPERIMENTAL DETAILS

We noticed that HydraMap's prediction package might have some performance issue on their 'holo mode' since it was producing nearly random outputs, and most of the predictions clashed with protein atoms. Thus their 'apo mode' is used instead, with all ligand molecules merged into the receptor PDB file. Our experiments showed that this configuration performed better, so all results of HydraMap are produced likewise.

For experiments using WATsite and GAsol, we followed strictly with their online manual (`https://pharma.unibas.ch/fileadmin/user_upload/pharma/Research_groups/Computational_Pharmacy/Bilder/Research/WATsite3_0_User_Guide_w_cudagl_docker_image.pdf`, last accessed on Jul. 31st, 2021 (Version Jan. 15th, 2020, Figure 6.1)). For WATsite, ater molecules that are within 6.0Å of proteins are generated, instead of the default cutoff (3.0Å), to comply with our evaluation standards.

OppA protein structures are submitted via the GalaxyWEB server to yield predictions from the GalaxyWater-wKGB model(`http://galaxy.seoklab.org/cgi-bin/submit.cgi?type=WKGB`).

In experiments, the definition of binding site ground truth waters are crystallographic waters within 4.0Å of the binding site (which was also used in HydraMap's experiment), and all predictions within 5.0Å of both protein and binding ligands are included in the evaluation.

WATsite3.0 User Guide
A GPU-accelerated Hydration Site
Prediction Program with PyMOL Plugin

Ying Yang, Matthew R. Masters, Amr H. Mahmoud,
Bingjie Hu, Markus A. Lill.

Department of Medicinal Chemistry and Molecular Pharmacology
College of Pharmacy, Purdue University
575 Stadium Mall Drive
West Lafayette, IN 47907
Email: mlill@purdue.edu
http://people.pharmacy.purdue.edu/~mlill

January 15, 2020

Figure 6.1. Manual file of the WATsite and GAsol package used in our experiment, first page.
